# Correction to: Update on human genetic susceptibility to COVID-19: susceptibility to virus and response

**DOI:** 10.1186/s40246-021-00360-1

**Published:** 2021-09-18

**Authors:** Vito Luigi Colona, Vasilis Vasiliou, Jessica Watt, Giuseppe Novelli, Juergen K. V. Reichardt

**Affiliations:** 1 Department of Biomedicine and Prevention, “Tor Vergata” University of Rome, 00133 Rome, Italy; 2 Department of Environmental Health Sciences, School of Public Health, Yale University, New Haven, USA; 3 College of Public Health, Medical and Veterinary Sciences, James Cook University, Smithfeld, QLD Australia; 4 IRCCS Neuromed, Pozzilli, IS Italy; 5 Department of Pharmacology, School of Medicine, University of Nevada, Reno, NV 89557 USA; 6 Australian Institute of Tropical Health and Medicine, James Cook University, Smithfeld, QLD 4878 Australia

## Correction to: Hum Genomics (2021) 15:57 10.1186/s40246-021-00356-x

Following publication of the original article [1], the authors identified an error in the family name of the second author Vasilis Vasiliou.

The incorrect author name is: Vasilis Vasilou.

The correct author names is: Vasilis Vasiliou.

The author group has been updated above and the original article [1] has been corrected.
